# Hypnotism in Its Relation to Hysteria and to Psychasthenia

**Published:** 1907-11-09

**Authors:** Douglas Bryan

**Affiliations:** Spa House, Leicester.


### HYPNOTISM IN ITS RELATION TO HYSTERIA AND TO PSYCHASTHENIA.

Sir,—Will you kindly grant me a little space for a few remarks anent Dr. Purves Stewart's article on psychasthenia appearing in your journal for October 19? Dr. Stewart states there that "hypnosis and suggestion constitute an integral part of hysteria." I should like to ask what is meant by this peculiar statement, for surely by hypnosis is understood "the sleep which is induced in a person by means of hypnotism." What this has to do as constituting an integral part of hysteria I fail to see; perhaps Dr. Stewart can explain; if not, I think it would have been better for him to have obtained a correct knowledge of terms before making use of such a phrase.

Another paragraph in the article to which I wish to refer is that in which Dr. Stewart, in speaking of the treatment of psychasthenia, says that "hypnotism, whatever its value in hysteria, is useless in psychasthenia." It seems a pity that Dr. Stewart should not have acquainted himself with actual facts before making such a statement, for he condemns his own remarks, as he positively asserts that the treatment of this disease lies chiefly in psychotherapeutics, which are neither more nor less than treatment by suggestions. If Dr. Stewart knew more about hypnotism and its use as a therapeutic agent he would know that the results of such treatment are based upon suggestions given to the patient by the hypnotist during hypnotic sleep, which, acting upon the person's mind, bring about a cure. The reason for hypnotising the patient is that the person, being in a hypnotic state, is much more amenable to suggestion, and thereby the results are generally more rapid and certain. It is greatly to be deprecated that medical men should make such statements without giving full and logical reasons for so doing, as they are extremely misleading both to the medical profession and those of the general public who may chance to see them.

Yours faithfully,

Douglas Bryan, M.R.C.S., M.R.C.P.

Spa House, Leicester, October 20.

